# ΗΙF1α, EGR1 and SP1 co-regulate the erythropoietin receptor expression under hypoxia: an essential role in the growth of non-small cell lung cancer cells

**DOI:** 10.1186/s12964-019-0458-8

**Published:** 2019-11-21

**Authors:** Tianhong Su, Pi Liu, Xinyu Ti, Shouzhen Wu, Xiaochang Xue, Zenglu Wang, Elhardji Dioum, Qiuyang Zhang

**Affiliations:** 1 Department of Medicine, Center for Esophageal Diseases, Baylor University Medical Center and Center for Esophageal Research, Baylor Scott & White Research Institute, 2 Hoblitzelle, Suite 252, 3500 Gaston Avenue, Dallas, TX 75246 USA; 2 Department of Liver Surgery, the First Affiliated Hospital of Sun Yat-sen University, Guangzhou, 510080 Guangdong China; 3 Department of Gastroenterology, the First Affiliated Hospital of Nanchang University, Nanchang, 330006 Jiangxi China; 4 Department of Respiratory Medicine, the Fourth Military Medical University, Xi’an, 710032 China; 5 Shaanxi Institute of Pediatric Diseases, Xi’an Children’s Hospital, Xi’an, 710003 China; 6 State Key Laboratory of Cancer Biology, Biotechnology Center, School of Pharmacy, The Fourth Military Medical University, Xi’an, 710032 China; 7 Department of Pharmacology, Department of Internal Medicine, the University of Texas Southwestern Medical School, Dallas, TX 75390 USA; 8 Present Address: Key Laboratory of the Ministry of Education for Medicinal Resources and Natural Pharmaceutical Chemistry, National Engineering Laboratory for Resource Development of Endangered Crude Drugs in Northwestern China, College of Life Sciences, Shaanxi Normal University, Xi’an, China; 9 Present Address: Diabetes Department, Nestle Institute of Health Science, EPFL Campus, 1015 Lausanne, Switzerland

**Keywords:** NSCLC, Hypoxia, EPO-R

## Abstract

**Background:**

Overexpression of erythropoietin (EPO) and EPO receptor (EPO-R) is associated with poor prognosis in non-small-cell lung carcinoma (NSCLC). Hypoxia, a potent EPO inducer, is a major stimulating factor in the growth of solid tumors. However, how EPO-R expression is regulated under hypoxia is largely unknown.

**Methods:**

The role of EPO-R in NSCLC cell proliferation was assessed by RNA interference in vitro. Luciferase reporter assays were performed to map the promoter elements involved in the EPO-R mRNA transcription. Nuclear co-immunoprecipitation and chromatin immunoprecipitation were performed to assess the interaction among transcription factors HIF1α, SP1, and EGR1 in the regulation of EPO-R under hypoxia. The expression of key EPO-R transcription factors in clinical specimens were determined by immunohistochemistry.

**Results:**

Hypoxia induced a dosage and time dependent EPO-R mRNA expression in NSCLC cells. Knockdown of EPO-R reduced NSCLC cell growth under hypoxia (*P* < 0.05). Mechanistically, a SP1-EGR1 overlapped DNA binding sequence was essential to the hypoxia induced EPO-R transcription. In the early phase of hypoxia, HIF1α interacted with EGR1 that negatively regulated EPO-R. With the exit of EGR1 in late phase, HIF1α positively regulated EPO-R expression through additive interaction with SP1. In clinical NSCLC specimen, SP1 was positively while EGR1 was negatively associated with active EPO-R expression (*P* < 0.05).

**Conclusions:**

HIF1α, SP1 and EGR1 mediated EPO-R expression played an essential role in hypoxia-induced NSCLC cell proliferation. Our study presents a novel mechanism of EPO-R regulation in the tumor cells, which may provide information support for NSCLC diagnosis and treatment.

**Graphical abstract:**

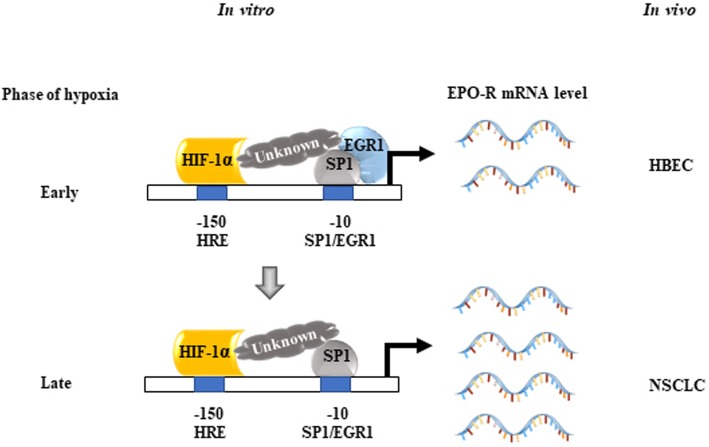

## Background

The solid tumor expansion is usually characterized by the existence of focal hypoxic regions which leave portions of the tumor suffering from oxygen deprivation. Although the hypoxic microenvironment may suppress tumor cells division or even lead to their death, it can also lead to alteration of metabolism in tumor cells to improve their chance for survival. Thus, hypoxia represents a paradox for various tumor studies. Accumulating evidence demonstrated that hypoxia has significant impacts on the behavior of a wide spectrum of tumors including non-small cell lung cancer (NSCLC) [1, 2]. Under hypoxic conditions, NSCLC is often educated to be more aggressive and prone to be radio- and chemo-resistant [3, 4]. Hypoxia-inducible factor 1 alpha (HIF1α) is one of the most potent factors that are widely linked to the behavior changes of hypoxic tumor cells [5]. HIF1α activates the transcription of dozens of genes including erythropoietin (EPO), which provide tumor cells with the device to maintain vigorous growth and expansion in a hypoxic microenvironment [6].

As a pleiotropic cytokine, EPO regulates bone marrow-derived erythroid progenitor proliferation, differentiation and survival via binding to erythropoietin receptor (EPO-R). It is well known that EPO-R is mainly expressed in erythroid, megakaryocytic and mast cells and the hematopoietic-specific transcription factor GATA-1 plays a pivotal role in the activation of the EPO-R promoter [7]. However, EPO-R is also found expressed in endothelial cells and brain [8–10]. In addition, recombinant EPO or erythropoiesis-stimulating agents (ESAs) can accidentally stimulate the growth of EPO-R-positive tumors when used for treating tumor-related anemia suggesting the universality and importance of tumor-associated EPO-R expression [11–15]. Like EPO, EPO-R expression is also dynamically regulated under hypoxic stress. The enhanced EPO signaling is found within hypoxic tumor regions with highest levels of EPO-R expression [16]. However, unlike EPO, the mechanism of hypoxia-mediated EPO-R expression is not delineated.

We previously reported that hypoxia can induce EPO expression and promote cell proliferation in NSCLC [17]. In the present study, we aim to investigate if and how hypoxia regulates EPO-R expression in NSCLC, and to determine if the transcription regulation of EPO-R has clinical relevance in NSCLC.

## Materials and methods

### Clinical specimen

Patient tumor and control tissue specimen were obtained from the First Affiliated Hospital of Sun Yat-sen University with written informed consents. In total, 20 patients who had surgical resection in 2006 were enrolled: 15 NSCLC and 5 lung bullae patients as control samples (Additional file 1: Supplementary Materials and Methods).

### Cell lines

Three normal human bronchial epithelial cells (HBEC-3KT, −4KT, and-6KT), six NSCLC cell lines (A549, H44, H2073, H1819, H1833, H3122), and one human EPO-dependent erythroleukemia line OCIM-1 were used in this study (Additional file 1: Supplementary Materials and Methods).

### Hypoxic treatment

Detailed is described in Additional file 1: Supplementary Materials and Methods.

### RNA extraction, real-time PCR, protein extraction and immunoblots

Detailed is described in Additional file 1: Supplementary Materials and Methods and in Additional file 2: Supplementary Tables.

### DNA constructs

The methods for construction of wild-type and site-specific mutation of human EPO-R promoters, and for cloning full-length or truncated, and wildtype or site-specific modified cDNA of the transcription factors are described in Additional file 1: Supplementary Materials and Methods.

### Construction of stable cell line using lentiviral particles

Detailed is described in Additional file 1: Supplementary Materials and Methods.

### Nuclear protein complex co-immunoprecipitation (co-IP) and chromatin immunoprecipitation (ChIP) assays

Nuclear co-IP was performed to evaluate the interaction among HIF1α, SP1 and EGR1 and ChIP to assess the binding activity of SP1 and/or EGR1 to proximal EPO-R promoter under hypoxia in A549 cells (Additional file 1: Supplementary Materials and Methods; Additional file 2: Supplementary Tables).

### Immunohistochemistry (IHC)

Details are described in Additional file 1: Supplementary Materials and Methods and listed antibodies are in Additional file 2: Supplementary Tables.

### Luciferase reporter assay

Luciferase reporter assays were done to characterize the EPO-R promoters as described previously [18] (Additional file 1: Supplementary Materials and Methods).

### Data analysis

Statistical analyses were performed using an unpaired Student’s t-test with the InStat for Windows statistical software package (GraphPad Software, San Diego, CA). For multiple comparisons, an ANOVA and the Student-Newman-Keuls multiple-comparisons test was performed using the InStat for Windows statistical software package (GraphPad). Differences were considered significant at *P* ≤ 0.05 (Additional file 1: Supplementary Materials and Methods).

## Results

### Hypoxia-induced EPO-R is essential to NSCLC cell growth

**Fig. 1:**
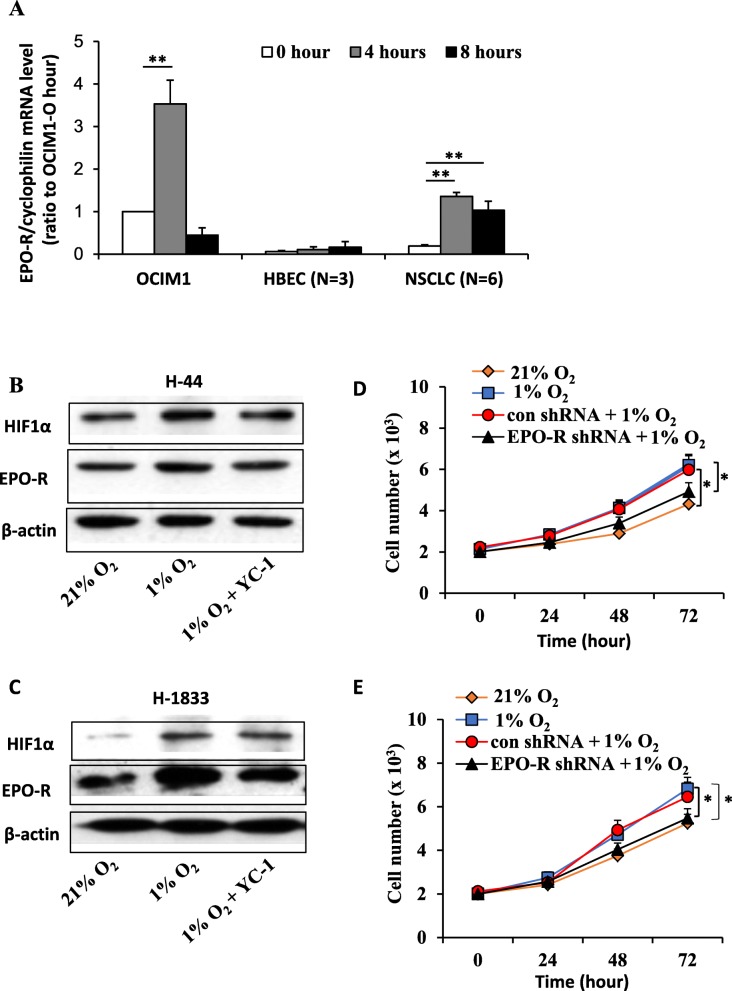
Hypoxia-induced EPO-R overexpression promotes cell proliferation in NSCLC. **a** Hypoxia induced EPO-R expression in NSCLC cells. Three HBEC (HBEC3KT, HBEC4KT and HBEC6KT) and 6 NSCLC (A549, H44, H2073, H1819, H1833, H3122) cell lines were treated with 1% hypoxia or room air for 0, 4 or 8 h. The erythroleukemia line OCIM-1 was used as a positive control. The mRNA level was determined by real-time RT-PCR with cyclophilin as an internal control. Mean ± SEM; ** *P* < 0.01. **b** and **c** Expression of EPO-R protein was upregulated under hypoxia in H44 (**b**) and H1833 cells (**c**), which was diminished by treatment with HIF1α inhibitor YC-1. Protein expression was determined by Western blots and β-actin was used as a loading control. **d** and **e** MTT assays showed that hypoxic treatment promoted H44 (**d**) and H1833 (**e**) cell growth, and knockdown of EPO-R with specific shRNA abolished these effects. The data are representative of three experiments. Mean ± SEM; *P < 0.05

The upregulation of EPO-R reported in various solid tumors has raised safety concerns for the use of EPO or ESAs to treat anemia in cancer patients. To investigate the potential role of EPO-R in NSCLC, we examined EPO-R protein and mRNA expression in 6 NSCLC and 3 HBEC cell lines with the high-EPO-R expressing erythroid cell line OCIM1 as a positive control [19]. EPO-R was significantly overexpressed in NSCLC cell lines as compared with those of HBEC lines (Additional file 3: Figure S1A and S1B). Hypoxia potently induced stable EPO-R overexpression in NSCLC cells (*P* < 0.05) (Fig. 1a). In addition, we found that the hypoxic inducibility was not different among the high and low-EPOR expressing NSCLC cells (data not shown). In OCIM1 cell, EPO-R was induced after 4 h treatment and returned to basal level 8 h later. Modest induction was found in HBEC cells (Fig. 1a).

Next, we used two representative cell lines H44 (low EPO-R expression) and H1833 (high EPO-R expression) to determine if hypoxia-induced EPO-R expression played a role in NSCLC cells. We first verified that hypoxia did increase EPO-R protein expression (Fig. 1b and c). HIF1α is the most potent transcription factor that mediated the hypoxic response in mammalian cells. As shown in Fig. 1b and c, hypoxia induced EPO-R protein expression which was inhibited by pretreating cells with a HIF1α inhibitor YC-1. By using the cells with stable knockdown of EPO-R, we found that EPO-R silencing almost completely abrogated hypoxia-induced cell proliferation (Fig. 1d and e). These data suggested that EPO-R was essential to promote NSCLC proliferation under hypoxia condition, independent of basal expression level of EPO-R.

### HIF1α and SP1 positively, whereas EGR1 negatively mediated hypoxia induced EPO-R mRNA transcription

To investigate how EPO-R was induced under hypoxic condition, we analyzed the − 200 bp proximal promoter region of EPO-R and identified several putative transcription factors binding sites including those for HIF (called hypoxia-responsive element, or HRE), SP1 and EGR1 (Additional file 3: Figure S2A). Among them, the overlapping SP1 and EGR1 site was found conservative among human, mouse and dog (Additional file 3: Figure S2B).

**Fig. 2:**
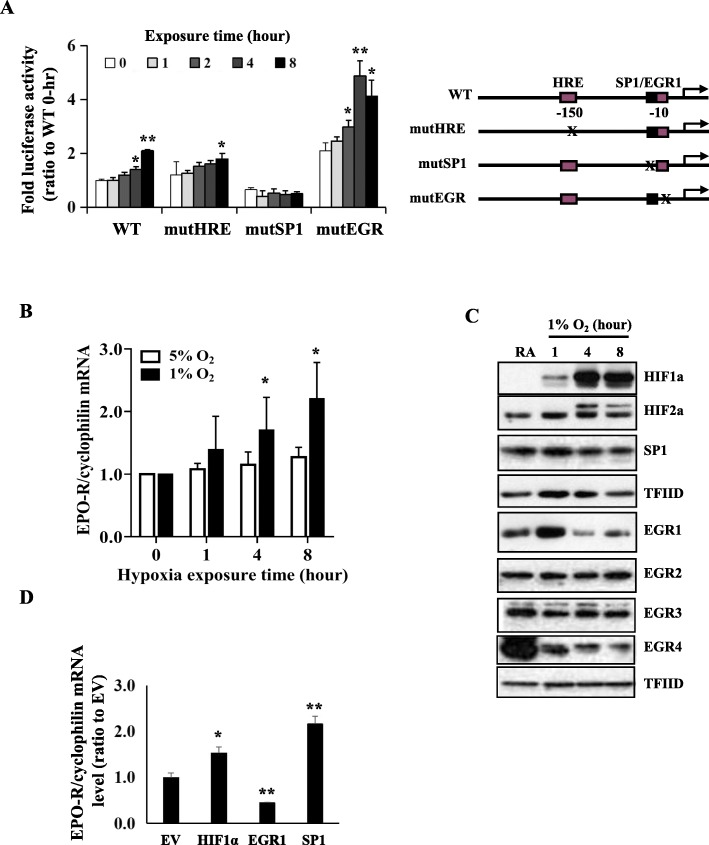
Identification of transcription factors that mediated EPO-R mRNA expression under hypoxia in A549 cells. **a** Luciferase reporter activity of the human EPO-R proximal promoters of wild-type, site-direct mutated HRE, SP1, and EGR1 sites under hypoxia. Mean ± SD; * *P* < 0.05, ** *P* < 0.01 versus “0” hour. **b** Time and dosage-dependent expression of EPO-R mRNA under hypoxia. mRNA was determined by real-time RT-PCR with cyclophilin as an internal control. Mean ± SEM; *P < 0.05 versus “0” hour. **c** Time–dependent nuclear protein expression of transcription factor HIF1α, HIF2α, SP1, EGR1, EGR2, EGR3 and EGR4 under hypoxia. Nuclear protein level was determined by Western blots with TGFIID included as a loading control. **d** Effects of HIF1α, SP1 and EGR1 overexpression on EPO-R mRNA expression. All the data were repeated three times. Mean ± SD; * *P* < 0.05, ** *P* < 0.01 versus empty vector (EV)

Next, we determined the role of these sequences on EPO-R expression under hypoxia in A549 cells. As expected, hypoxia induced gradual increase of wild-type EPO-R promoter activity (Fig. 2a). With point mutated SP1 and intact EGR1 sites, basal promoter activity was decreased and hypoxic inducibility was diminished (Fig. 2a). On the other hand, with point mutated EGR1 and intact SP1 sites, the basal promoter activity was increased and hypoxic inducibility was maintained (Fig. 2a). These data suggested that EPO-R mRNA transcription was positively regulated by SP1 but negatively regulated by EGR1. Surprisingly, the abolishment of putative HRE site did not affect hypoxic inducibility of EPO-R promoter (Fig. 2a). These data also suggested that A549 is a valid NSCLC cell line for studying mechanism of hypoxia mediated transcription of EPO-R in NSCLC.

Next, we determined if these transcription factors were involved in hypoxia inducible expression of EPO-R. As expected, hypoxia induced EPO-R mRNA expression in dose- and time-dependent fashion in A549 cells (Fig. 2b). Correspondingly, exposure to 1% O_2_ resulted in gradual increase of nuclear HIF1α during the 8 h treatment (Fig. 2c). Hypoxia induced a sharp increase of nuclear EGR1 at 1 h followed by rapid decline to lower than baseline level (Fig. 2c). Modest or no changes were found in HIF2α, SP1, EGR2 and EGR3 and decrease was found in EGR4 expression levels (Fig. 2c). We then measured EPO-R mRNA in A549 cells with ectopic expression of HIF1α, SP1 or EGR1 which were responsive to hypoxia treatment and/or has a binding site within the proximal EPO-R promoter. As shown in Fig. 2d, HIF1α and SP1 promoted whereas EGR1 inhibited EPO-R expression, confirming that HIF1α, SP1 and EGR1 played a role on the hypoxia-mediated EPO-R gene transcription. Again, these data further confirmed that A549 was feasible as a model cell line for mechanistic study of hypoxia-mediated EPO-R regulation.

### The interaction of HIF1α and SP1 promoted EPO-R transcription under hypoxia

**Fig. 3:**
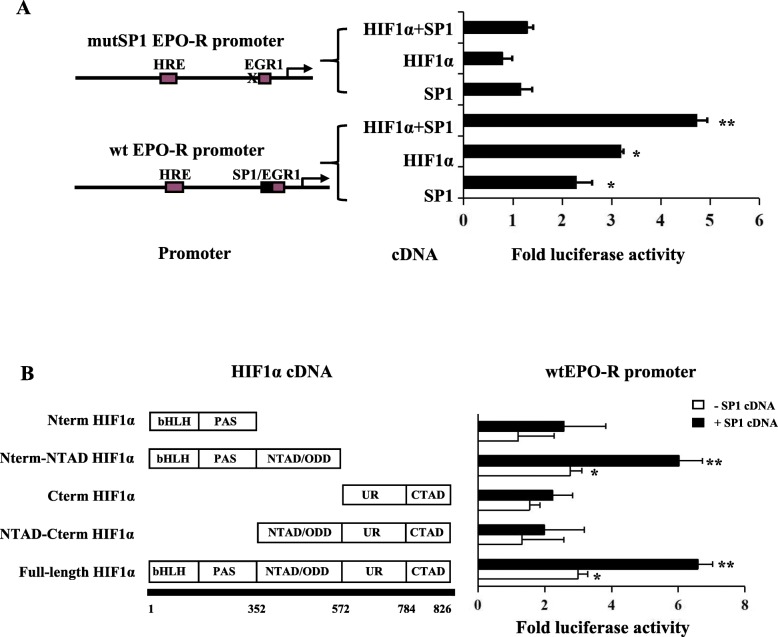
Synergistic interaction between HIF1α and SP1 promoted EPO-R transcription under hypoxia. **a** Luciferase reporter activity of the EPO-R proximal promoters of wild-type or site-direct mutated SP1 site with co-expression of HIF1α and SP1 cDNA alone or HIF1α and SP1 cDNA in combination. **b** Luciferase reporter activity of the wild-type EPO-R proximal promoters with co-expression of full-length or truncated HIF1α alone or in combination with SP1 cDNA. The empty cDNA and empty promoter vectors were included as negative controls (EV). The fold activity was calculated first by normalizing to cDNA EV and then to promoter EV. Data are from three independent repeats and are represented as Mean ± SD; * *P* < 0.05, ** *P* < 0.01 versus empty vector

Since the HRE site was not essential to hypoxia mediated EPO-R transcription, we speculated that HIF1α promotes EPO-R expression through interacting with SP1 under hypoxia. We transfected A549 cells with a wild-type (wt) or SP1 site mutated EPO-R luciferase promoter, together with HIF1α and/or SP1 cDNA. As expected, SP1 and HIF1α alone significantly increased wt EPO-R promoter activity (Fig. 3a). The combination of HIF1α and SP1 cDNA further increased wt EPO-R promoter activity additively (Fig. 3a). In the SP1 site mutated promoter, neither SP1 and HIF1α each alone nor in combination enhanced the promoter activity (Fig. 3a). These data suggested that HIF1α and SP1 regulated EPO-R through interacting with the SP1 binding sequence.

To elucidate how HIF1α interacts with SP1 to regulate EPO-R expression, a series of truncated HIF1α cDNAs were co-transfected with wild type EPO-R promoter into A549 cells, in the absence or presence of SP1 cDNA. As expected, the full-length HIF1α positively interacts with SP1 to promote EPO-R promoter activity (Fig. 3b). The HIF1α fragment without the unique region (UR) and C-terminal transactivation domain (CTAD) did not affect its interaction with SP1 for this regulation (Fig. 3b). However, the HIF1α fragment without basic helix–loop–helix (bHLH) and PER-ARNT-SIM (PAS) or N-terminal transactivation (NTAD) domains lost its basal activity and its capacity to interact with SP1 (Fig. 3b). All these data indicated that the bHLH, PAS and NTAD domains are required for HIF1α and SP1 interaction in EPO-R regulation under hypoxia condition.

### EGR1 inhibited EPO-R mRNA transcription through negatively regulating the HIF1α and SP1 interaction

**Fig. 4:**
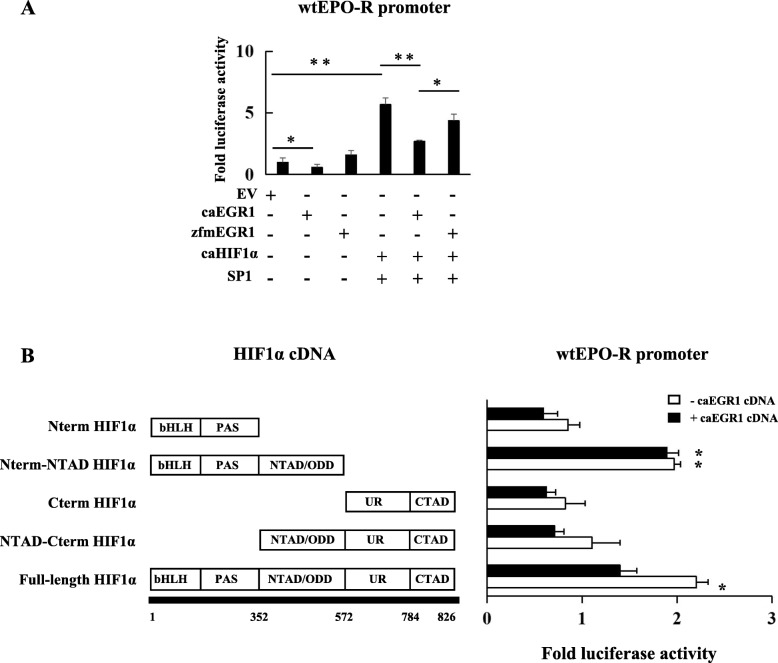
EGR1 inhibited EPO-R transcription by negatively interacting with HIF1α under hypoxia. **a** Luciferase reporter activity of the wild-type EPO-R proximal promoters with co-expression of empty vector, constitutive active (ca) EGR1, or zinc-finger mutated (zfm) EGR1 cDNAs alone, or with caHIF1α and SP1, caHIF1α and SP1 and caEGR1, caHIF1α and SP1 and zfmEGR1 in combination. **b** Luciferase reporter activity of the wild-type EPO-R proximal promoters with co-expression of full-length or truncated HIF1α together with or without wild-type EGR1 cDNA. The empty cDNA and empty promoter vectors were included as controls. The fold change of luciferase activity was calculated first by normalizing to cDNA EV and then to promoter EV. Data are from three independent repeats and are represented as Mean ± SD; * *P* < 0.05 and **P < 0.01 versus empty vector

As a member of the zinc finger transcription factor family, EGR1 has been widely reported to serve as a tumor suppressor gene in various tumors [20]. We identified an EGR1 binding site in the EPO-R promoter and deletion of this site potently stimulated EPO-R promoter activation (see Fig. 2a). In addition, EGR1 was induced at the early stage of hypoxia and then declined rapidly (see Fig. 2c), which led us to speculate that EGR1 may negatively regulates EPO-R. To test this hypothesis, we transfected A549 cells with the wt EPO-R promoter in combination with a constitutively active (ca) EGR1 (caEGR1) or with a zinc-finger domain mutated EGR1 (zfmEGR1) cDNA. The caEGR1 significantly suppressed EPO-R promoter activity (Fig. 4a) while the zfmEGR1 activated EPO-R promoter activity (Fig. 4a), suggesting that DNA binding was essential to the inhibition of EPO-R transcription by EGR1. Next, we transfected A549 cells with a wild-type (wt) EPO-R luciferase promoter, together with HIF1α, SP1, and caEGR1 or zfmEGR1 cDNA. The caEGR1 eliminated the synergistic interaction between HIF1α and SP1 while zfmEGR1 partially restored this interaction (Fig. 4a) (*P* < 0.05).

Next, we tested whether EGR1 can negatively interact with HIF1α to regulate EPO-R transcription. As shown in Fig. 4b, EGR1 significantly suppressed the full-length HIF1α-induced wt EPO-R promoter activity. Results again showed that bHLH, PAS, NTAD/ODD domains were essential to the HIF1α basal activity, and the C-terminal regions of the UR and CTAD domains were required for a significant negative interaction between HIF1α and EGR1 (Fig. 4b).

### HIF1α regulated EPO-R through sequential interaction with EGR1 and SP1 under hypoxia

**Fig. 5:**
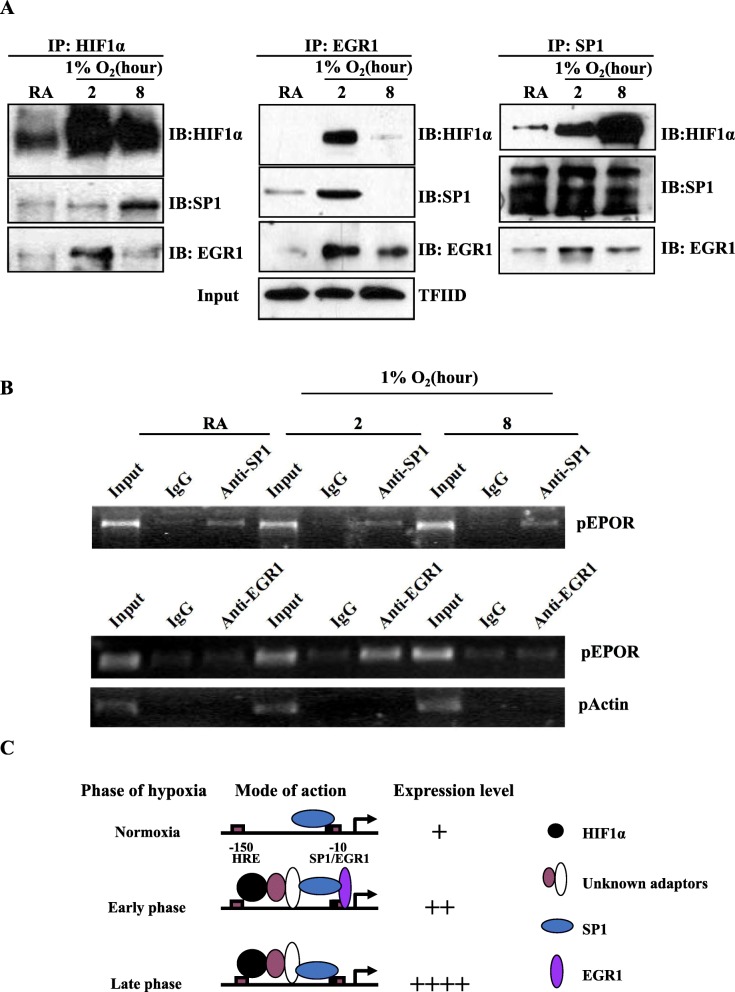
Immunoprecipitation analysis of the protein-protein and protein-DNA interactions around proximal EPO-R promoter under hypoxia. **a** Nuclear co-immunoprecipitation of the transcription factors HIF1α, EGR1, and SP1. Nuclear lysates were pulled down by immunoprecipitation (IP), followed by western blotting (IB) detection with TFIID used an input control. The experiments were repeated at least twice. **b** Chromatin immunoprecipitation (ChIP) analysis of promoter DNA binding activity under hypoxia. The chromatins pull-downed by antibody against SP1 or EGR1, or by normal IgG were analyzed by standard PCR using primers for proximal EPO-R promoter (pEPO-R). βactin promoter primers (pActin) were used as an internal control. The experiments were repeated at least twice. **c** A diagram depicts how HIF1α, EGR1, and SP1 cooperatively and sequentially regulates EPO-R expression under hypoxia condition in cultured NSCLC cells

Since the interaction between HIF1α and SP1 or EGR1 was critical for positive or negative regulation of EPO-R expression respectively, we next asked how these three transcription factors interact during hypoxic exposure. A sequential co-IP assay with nuclear extracts indicated that HIF1α interacted more with EGR1 at an earlier time point (2 h) but interacted with SP1 more at later time point (8 h) after hypoxic exposure (Fig. 5a); EGR1 interacted with HIF1α and SP1 both more at early time point (2 h) than late time point (8 h); SP1 interacted with HIF1α gradually increased from early to late time point (Fig. 5a).

As SP1 and EGR1 can each interact with the overlapping SP1-EGR binding site of the EPO-R promoter, we next determined if these two proteins physically bind to this DNA sequence under hypoxia. As shown in Fig. 5b, SP1 was constitutively bound to the EPO-R promoter which was not changed by hypoxia. However, binding by EGR1 was induced at the early time point of hypoxia but returned to basal level at later time point (Fig. 5b).

The aforementioned data assisted us to draw a diagram that summarizes how HIF1α, EGR1, and SP1 cooperatively and sequentially regulate EPO-R expression in response to hypoxia in vitro (Fig. 5c). Under normoxia, EPO-R expresses at a low level which is maintained by SP1. EPO-R expresses slightly higher at the early phase of hypoxia when EPO-R promoter was concurrently bound by EGR1, HIF1α and SP1. EGR1 counteracts the upregulation effects by HIF1α and SP1. At the late phase of hypoxia, EPO-R was potently induced when EGR1 expression level declined and the synergistic effect of HIF1α and SP1 was enhanced. Taken together, the additive and sequential interactions of HIF1α, EGR1 and SP1 finely mediate EPO-R expression in NSCLC under hypoxia, which affects NSCLC progression and makes it response sensitively to the tumor microenvironment.

### SP1 was upregulated and EGR1 downregulated in NSCLC

**Fig. 6:**
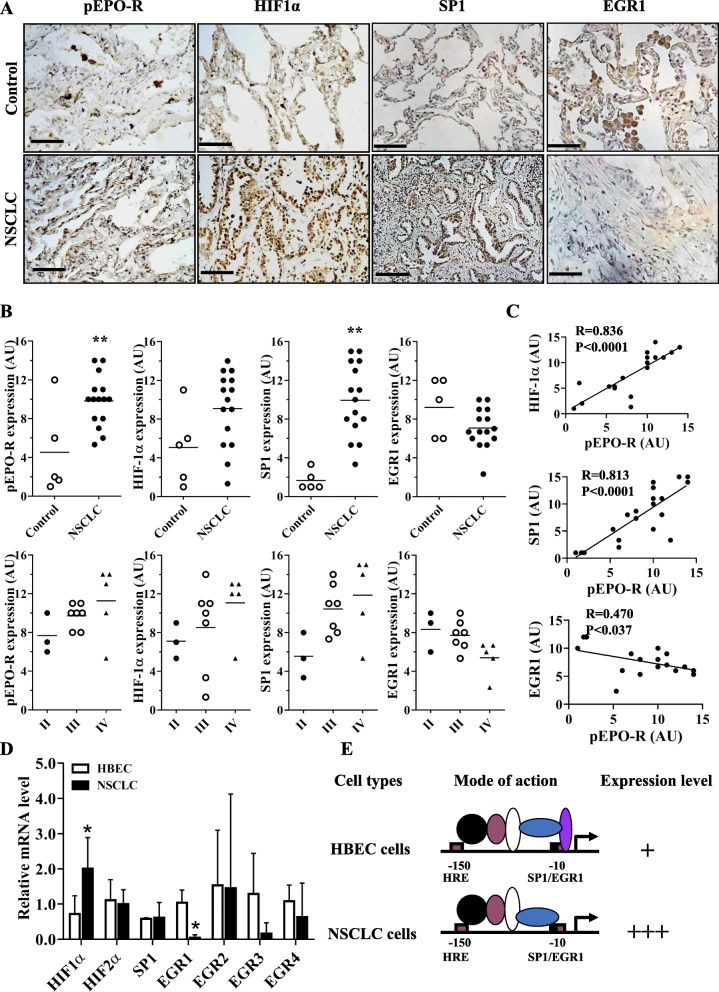
Expressions of phosphor-EPO-R (pEPO-R), HIF1α, SP1 and EGR1 in the NSCLC clinical specimens. **a** Immunohistochemical staining and scoring **b** of pEPO-R, HIF1α, SP1 and EGR1 in clinical NSCLC specimens or normal controls. Bars = 50 μM. **c** Correlation analysis of HIF1α, SP1 and EGR1 with pEPO-R expression in NSCLC. **d** RT-PCR analysis of HIF1α, HIF2α, SP1 and EGR1–4 mRNA expression in the NSCLC cell lines under normoxic condition. Mean ± SD; *, *P* < 0.05. **e** A diagram depicts how HIF1α, EGR1, and SP1 regulate EPO-R expression under normoxic condition in HBEC and NSCLC

Considering that EGR1 and SP1 play pivotal roles on EPO-R expression, we examined their levels in 15 cases of NSCLC and 5 cases of normal lung specimens by immunohistochemistry (Additional file 2: Table S3). As shown in Fig. 6a, b and Additional file 2: Table S4, SP1 was upregulated whereas EGR1 was downregulated in the NSCLC specimens as compared with the normal lung tissues. We also found that phosphor-EPO-R (pEPO-R) and HIF1α were also significantly higher in NSCLC, which is consistently with our previous report [17]. The expression levels of pEPO-R, HIF1α and SP1 were positively while EGR1 was negatively associated in NSCLC (Fig. 6c). In the 6 NSCLC and 3 HBEC cell lines used in this study, we found HIF1α was significantly higher while EGR1 was significantly lower in NSCLC cells compared to HBEC cells (*P* < 0.05) (Fig. 6d). Based on these data, we again hypothesized that it is HIF1α, EGR1 and SP1 that govern EPO-R expression in HBEC and NSCLC cells (Fig. 6e). All these data collectively supported our in vitro and in vivo findings and suggested that HIF1α, EGR1 and SP1 are critical factors that can be induced by hypoxia to control the EPO-R expression in the progression of NSCLC.

## Discussion

The role of EPO signaling on tumor growth remains unclear nowadays, which is partly due to undefined EPO-R expression pattern in tumor cells. On one hand, EPO-R can be overexpressed and play critical roles in tumor progression such as those associated with post ESA/rhEPO treatment [11–15]. On the other hand, no significant EPO-R expression was detected in several systematic screenings in both tumor cell lines and solid tumor specimens [21, 22]. In NSCLC, we previously identified high- and low-EPO-R cell population and we found that tumor-derived EPO significantly stimulated the growth of EPO-R-positive NSCLC cells [17]. Here in this study, we further confirmed that EPO-R expression was inducible under hypoxia (independent of basal expression level) in NSCLC.

Unlike EPO, the transcriptional regulation of EPO-R under hypoxia is not well studied. Several transcription factors including GATA-1 and SP1 have been identified to directly regulate the EPO-R mRNA expression but how these factors are linked to hypoxia-mediated EPO-R expression remain unknown [7, 23]. In the present study, we identified a cis-acting DNA element essential for hypoxia induced expression of human EPO-R for the first time. We found that the sequential interactions between HIF1α/EGR1 and HIF1α/SP1 that govern EPO-R expression at different phases of hypoxic exposure. Our future studies will focus on whether other transcription factors also participated in the transcriptional regulation complex of EPO-R promoter.

EGR1 is a prototypical member of the zinc-finger transcription factors family and a range of molecular and environmental stimuli and stressors have been reported to induce EGR1 expression. EGR1 regulates the expression of dozens of target genes such as insulin-like growth factor-II (IGF-II) [24], BCL-2 [25], PTEN [26, 27] and tumor necrosis factor-alpha (TNFα) [28]. As a result, EGR1 exerts contradictory activities by modulating different signaling pathways. In NSCLC, EGR1 is reported to inhibit its malignancy and development by regulating KRT18 [29]. Here, we reported that the induction of EGR1 by hypoxia plays a negative role on EPO-R expression in NSCLC. In addition, very low level of EGR1 can be detected both in NSCLC cell lines and in clinical specimens as compared with the normal controls. All these data collectively suggested that EGR1 acts as a suppressor gene in NSCLC.

SP1 is another zinc-finger transcription factor that can regulate gene expression through synergistically interacting with EGR1 such as that in PDGF [30]. Conversely, in this study we found that SP1 and EGR1 bind competitively to an overlapping DNA element in the proximal human EPO-R promoter and consequently, counteract to regulate its transcription. We found that hypoxia did not alter nuclear SP1 protein level, which suggest that the major contribution of SP1 to EPO-R regulation are through increased binding to EPO-R promoter to promote recruitment of hypoxia inducible factors such as HIF1α.

## Conclusions

EPO-R expression is essential to NSCLC cell growth under hypoxic condition. Hypoxia induced EPO-R is mediated by HIF1α through sequential interaction with EGR1 and SP1; thus, the hypoxia/HIF1α/EGR1/SP1/EPO-R axis may be potential targets for NSCLC diagnosis and therapy.

## Supplementary information

**Additional file 1.** Supplementary Materials and Methods.

**Additional file 2: Table S1.** The oligonucleotide primers used in this study. **Table S2.** A list of antibodies used in this study. **Table S3.** Patient characteristics. **Table S4.** Correlation between SP1/EGR1 expression and clinicopathological characteristics in patients with NSCLC.

**Additional file 3: Figure S1.** EPO-R protein (A) and mRNA (B) were expressed higher under normoxia in NSCLC cells. **Figure S2.** Identification of a cis-DNA elements dictated EPO-R regulation under hypoxia in NSCLC cells.

## Data Availability

All data developed or analyzed during this study are presented either in this article or in the Supplementary Materials and Methods, Tables, Figures and Figure Legends files.

## Abbreviations

ChIP: Chromatin immunoprecipitation
EGR1: Early growth response-1
EPO: Erythropoietin
EPO-R: Erythropoietin receptor
HBEC: Human bronchial epithelial cell
HIF1α: Hypoxia-inducible factor 1-alpha
HRE: Hypoxia-responsive element
NSCLC: Non–small cell lung cancer
SP1: Specificity protein 1
