# Supplementary material for: ΗΙF1α, EGR1 and SP1 co-regulate the erythropoietin receptor expression under hypoxia: an essential role in the growth of non-small cell lung cancer cells
Source: Cell Commun Signal. 2019 Nov 21;17:152. doi: 10.1186/s12964-019-0458-8 (PMC6869211; doi:10.1186/s12964-019-0458-8)
Supplement: Supplementary file 1 — Additional file 1. Supplementary Materials and Methods. [file 12964_2019_458_MOESM1_ESM.docx]

**Additional File**[**1**](https://molecular-cancer.biomedcentral.com/articles/10.1186/s12943-019-1031-1#MOESM1)**: Supplementary Materials and Methods.**

**Materials and methods**

**Clinical specimen**

Patient tumor and control tissue specimen were obtained from the First Affiliated Hospital of Sun Yat-sen University with written informed consents. In total, 20 patients who had surgical resection in 2006 were enrolled: 15 NSCLC and 5 lung bullae patients as control samples. The 15 patients were histologically confirmed to have NSCLC staging from I A to IV A according to the WHO criteria and the tumor-node-metastasis classification. None of the patients had ever received neoadjuvant chemotherapy before surgery. This study strictly followed the institutional review board–approved protocols from the First Affiliated Hospital of Sun Yat-sen University, following the ethical guidelines of the 1975 Declaration of Helsinki.

**Cell lines**

Normal human bronchial epithelial cells (HBEC-3KT, -4KT, and-6KT) and NSCLC cell lines (H44, H2073, H1819, H1833, H3122) were provided by Dr. John Minna (University of Texas Southwestern Medical Center, Dallas, TX). NSCLC cell line A549 was purchased from the American Type Culture Collection (Manassas, VA). The human EPO-dependent erythroleukemia line OCIM-1 was provided by Dr. Broudy (University of Washington, Seattle). All cell lines were authenticated by short tandem repeat profiling (Microread Genetics, Shanghai, China). HBEC cells were cultured in Keratinocyte-serum free medium with a supplementation of Epidermal Growth Factor, Bovine Pituitary Extract and gentamycin (GIBCO/Invitrogen, Carlsbad, CA). A549 were cultured in DMEM/F12(1:1) medium (GIBCO) supplemented with 10% fetal bovine serum (FBS) and antibiotics. Other cells were cultured in RPMI 1640 medium (GIBCO) with 10% FBS and antibiotics.

**Hypoxic treatment**

Cells were grown in 6-well plates to about 70% confluence prior to hypoxic treatment. Hypoxia treatments were performed at 37 °C in a humidified hypoxic chamber (Coy Laboratory Products, Inc, Grass Lake, MI) filled with 5% or 1% O_2_ and 5% CO_2_ and balanced with N_2_. Control cells were incubated at 37 °C under normoxic conditions in 21% O_2_ and 5% CO_2_. Cells were harvested after 1, 2, 4, or 8-hour treatment with controls included at each time point.

**RNA extraction and real-time PCR**

For each time point, triplicate wells of cells were processed for protein and total RNA assays. Total RNA was extracted using Qiagen RNA Easy kit included with on-column DNase Digestion. EPO-R, HIF1α, HIF2α , SP1, EGR1, EGR2, EGR3, and EGR4 mRNA levels were measured by real time PCR using an established protocol [1] and cyclophilin-A was used as the reference gene. The primers (Supplementary Table 1) were designed using Primer Express (Applied BioSystems, Foster City, CA) and manufactured by Integrated DNA Technologies (Coralville, IA).

**Protein extraction and immunoblots**

Nuclear and cytosolic proteins were prepared using NE-PER Nuclear and Cytosolic Extraction Reagents (cat# 78833, Pierce biotechnology, Rockford, IL) and quantified by Bradford assay (Bio-Rad Laboratories, Hercules, CA). Aliquots of 20 µg nuclear protein were resolved by SDS-PAGE and transferred onto polyvinylidene difluoride membranes. The blots were blocked in Blotto-Tween solution (5% nonfat dry milk, 0.05% Tween in PBS) for 1 hour and then incubated in Blotto-Tween with the primary antibodies (Supplementary Table 2) for 2 hours. For loading control, the membranes were stripped and re-probed with mouse anti-β-actin monoclonal antibody for total protein, or rabbit anti-TFIID antibody for nuclear protein (Supplementary Table 2). Labeled protein was visualized by using a chemiluminescence detection system (ECL, Amersham, Piscataway, NJ) and quantified by densitometry.

**DNA constructs**

EPO-R promoter was directionally cloned by PCR into pGL4 luciferase reporter vector (Promega, Madison, WI) using NheI and HindIII sites with primers 5’-GAAGCTAGCGTAAGGTAAGTCACCTGTC-3’ and 5’-CAGAAGCTTCTAAGTGGCAGATCC-3’. Point mutations to HIF1α, SP1, and EGR1 binding sites were prepared by QuikChange II Site-directed mutagenesis kit (Agilent, Santa Clara, CA). Human HIF1α, human SP1, mouse EGR1, EGR2, EGR3 and EGR4 full-length cDNAs were cloned into pIRES vector (Clontech laboratories, Inc., Moutainview, CA). Constitutive HIF1α with inactivated prolyl and asparagyl hydroxylation sites was generated by site-directed mutagenesis [1]. Constitutive mouse EGR1 (caEGR1, I290F) and zinc finger mutated mouse EGR1 (zfmEGR1, G400Y) were prepared by QuikChange II Site-directed mutagenesis kit. For HIF1α and SP1/EGR1 interaction study, full-length and series of truncated human HIF1α cDNAs were cloned into pcDNA3.1 (Invitrogen, Carlsbad, CA).

**Construction of stable cell line using lentiviral particles**

To establish stable cell lines with EPO-R knockdown, we purchased custom shRNA against human EPO-R from GeneCopoeia (Rockville, MD). Lentiviral particles were generated from this lentiviral shRNA construct together with packaging plasmids pMDL and pRSV-Rev and envelope plasmid pCMV-VSVG in HEK 293T cells. After transduction, cells were subjected to initial screening in a medium containing 2 μg/ml of puromycin. Individual clones were selected based on Western blot screening.

**Nuclear protein complex co-immunoprecipitation**

Nuclear extracts were prepared from ~1.0 x 10^7^ A549 cells using the Nuclear complex Co-IP Kit (Active Motif, Carlsbad, CA). After centrifuge at 4℃ for 5min, the nuclear proteins were pre-cleaned with 50% Protein A magnetic bead slurry (Cell Signaling Technology, CST, Beverly, MA). The pulldown antibodies (supplementary table 2) were added to 200 µl nuclear lysate at 1 µg/100 µg of input protein, and incubated with rotation overnight at 4°C. Protein A magnetic beads (10–30 µl of 50% bead slurry) were used to pull down the target proteins. The levels of SP1, HIF1α, and EGR1 in the pellet were analyzed by Western blot.

**Chromatin immunoprecipitation (ChIP) assay**

ChIP assay was performed on A549 cells using a mouse anti-SP1 antibody or anti-Egr1 antibody (Supplementary Table 2) based on an established protocol [2]. Normal rabbit or mouse IgG was used as a negative control. Two primer sets were designed to flank the overlapping SP1-EGR1 binding site in the promoter region of EPO-R and non-related actin promoter primers included as a negative control. The amount of the specific DNA fragment was then quantified by PCR and agarose gel electrophoresis.

**Immunohistochemistry (IHC)**

Immunochemical staining was performed on 5 μm-thick sections of FFPE specimens and tissue microarrays using the primary antibodies against phosphor-EPOR, HIF1α, SP1 or EGR1 (Supplementary Table 2). The staining procedure was based on our established procedure as reported previously [3]. The sections were counterstained with Harris' hematoxylin. The staining was evaluated blindly by two observers with a scoring system as follows: 0, negative expression; 1+, moderately positive expression; 2+, strongly positive expression.

**Luciferase reporter assay**

Luciferase reporter assays were done to characterize the EPO-R promoters as described previously [4]. In brief, A549 cells were transfected with the pGL4-EPO-R vector constructed with EPO-R promoter region as indicated, either alone or in combination with different transcription factors encoding cDNAs. The empty vector was used as the control group for luciferase activity normalization. Luciferase activity was measured 24 hours after transfection following hypoxic exposure, or 48 hours after co-transfection with HIF1α, SP1 and/or EGR1 cDNA.

**Data analysis**

For real-time PCR, triplicate assays were performed for each time point within an experiment and each experiment was repeated two times. The relative quantity of mRNA with respect to the reference gene cyclophilin was calculated as described previously [1] and the results expressed as a ratio to the mean value in the corresponding normoxia or empty vector controls. For reporter assays, luciferase activity of the EPO-R promoter was normalized to that of the empty vector control within each transfection set and were averaged from three repeated experiments and compared to that of corresponding controls. Statistical analyses were performed using an unpaired Student’s t-test with the Instat for Windows statistical software package (GraphPad Software, San Diego, CA). For multiple comparisons, an ANOVA and the Student-Newman-Keuls multiple-comparisons test was performed using the Instat for Windows statistical software package (GraphPad). Differences were considered significant at P ≤ 0.05.
