# Supplementary material for: ΗΙF1α, EGR1 and SP1 co-regulate the erythropoietin receptor expression under hypoxia: an essential role in the growth of non-small cell lung cancer cells
Source: Cell Commun Signal. 2019 Nov 21;17:152. doi: 10.1186/s12964-019-0458-8 (PMC6869211; doi:10.1186/s12964-019-0458-8)
Supplement: Supplementary file 2 — Additional file 2: Table S1. The oligonucleotide primers used in this study. Table S2. A list of antibodies used in this study. Table S3. Patient characteristics. Table S4. Correlation between SP1/EGR1 expression and clinicopathological characteristics in patients with NSCLC. [file 12964_2019_458_MOESM2_ESM.docx]

**Additional File 2: Supplementary Tables**

**Supplementary Table 1. The oligonucleotide primers used in this study**

| **Gene name** | **Upstream sequence** | **Downstream sequence** | **Application** |
| --- | --- | --- | --- |
| EPO-R | tggtatctgactctggcatctc | tccctgatcatctgcagcc | RT-PCR |
| HIF1α | ccagttacgttccttcgatcagt | tttgaggacttgcgctttca | RT-PCR |
| SP1 | ggtgccttttcacaggctc | cattgggtgactcaattctgct | RT-PCR |
| EGR1 | gccagtataggtgatggggg | acctgaccgcagagtcttttc | RT-PCR |
| EGR2 | tgaccatctttcccaatgc | atccaacgacctcttctctcc | RT-PCR |
| EGR3 | aactgcctgacaatctgtacc | gatgtccattacattctctgtagc | RT-PCR |
| EGR4 | agcgagttttccgaacccg | gagtcggctaagtccccact | RT-PCR |
| Cyclophilin | cccaccgtgttcttcgacat | ccagtgctcagagcacgaaa | RT-PCR |
| EPO-R promoter | tggaatgttcgctatgtgccagg | tcccgaccaggcgcctctaa | ChIP |
| β-actin promoter | tcctcctcttcctcaatctcg | aaggcaactttcggaacgg | ChIP |

All sequences shown are 5’ to 3’.

**Supplementary Table 2. A list of antibodies used in this study**

| **Antibody** | **Source information**  **(Vendor, Cat#)** | **Dilution** | **Application** |
| --- | --- | --- | --- |
| EPO-R | Santa cruz, sc-697 | 1:200 | WB |
| Phospho-EPO-R (pY485) | Epitomics, 2585-1 | 1:50 | IHC |
| β-actin | Sigma, A5441 | 1:3000 | WB |
| HIF1α | BD Biosciences, 610959 | 1:1000 | WB |
| HIF1α | Cell Signaling, 14179 | 1µg/100 µg total protein | Co-IP |
| HIF1α | Thermo Scientific, MS-1164 | 1:100 | IHC |
| HIF2α | Novus Biologicals, NB100-132 | 1:1000 | WB |
| SP1 | Santa Cruz, sc-59 | 1:200  1:100  1µg/100 µg total protein | WB  IHC  Co-IP, ChIP |
| EGR1 | Cell signaling, #4153 | 1:1000  1:50 | WB  IHC |
| EGR1 | Santa Cruz, sc-110 | 1µg/100 total protein | Co-IP, ChIP |
| EGR2 | Covance, PRB-236P | 1:500 | WB |
|  |  |  |  |
| EGR3 | Santa cruz, sc-191 | 1:200 | WB |
| EGR4 | Abcam, ab50636 | 1:1000 | WB |
| TFIID | sc-204, Santa Cruz | 1:3000 | WB |
| normal IgG | sc-2027 L, Santa Cruz | 6 µg/ml | ChIP |

**Supplementary table 3. Patient characteristics**

| **Characteristics** | **NSCLC patients**  **(n=15)** | **Control**  **(n=5)** |
| --- | --- | --- |
| Age (years)^a^ | 59 (17-80) | 49 (18-67) |
| Sex (male/female) | 6/9 | 5/0 |
| Stage (I/II/III/IV) | 0/3/7/5 | - |
| Histotype | Adenocarcinoma | - |
| Smoking habit (N/Y) | 13/2 | 4/1 |

^a^Median (range)

**Supplementary Table 4 Correlation between SP1/EGR1 expression and clinicopathological characteristics in patients with NSCLC.**

| **Characteristics** | **patients** | **SP1 expression** | | | | | **EGR1 expression** | | | |
| --- | --- | --- | --- | --- | --- | --- | --- | --- | --- | --- |
|  |  | - | + | ++ | +++ | | - | + | ++ | +++ |
| Gender |  |  |  |  |  | |  |  |  |  |
| Male | 6 |  | 1 | 1 | | 4 | 2 | 4 |  |  |
| Female | 9 |  |  |  | | 9 | 2 | 7 |  |  |
| Age (Years) |  |  |  |  | |  |  |  |  |  |
| <60 | 8 |  | 1 | 1 | | 6 | 4 | 4 |  |  |
| >=60 | 7 |  |  |  | | 7 |  | 7 |  |  |
| Stage |  |  |  |  | |  |  |  |  |  |
| I | 0 |  |  |  | |  |  |  |  |  |
| II | 3 |  |  | 1 | | 2 | 1 | 2 |  |  |
| III | 7 |  |  | 1 | | 6 | 1 | 6 |  |  |
| IV | 5 |  |  |  | | 5 | 2 | 3 |  |  |
| Normal control | 5 | 4 | 1 |  | |  |  |  | 2 | 3 |
|  |  |  |  |  | |  |  |  |  |  |
