# Supplementary material for: ΗΙF1α, EGR1 and SP1 co-regulate the erythropoietin receptor expression under hypoxia: an essential role in the growth of non-small cell lung cancer cells
Source: Cell Commun Signal. 2019 Nov 21;17:152. doi: 10.1186/s12964-019-0458-8 (PMC6869211; doi:10.1186/s12964-019-0458-8)
Supplement: Supplementary file 3 — Additional file 3: Figure S1. EPO-R protein (A) and mRNA (B) were expressed higher under normoxia in NSCLC cells. Figure S2. Identification of a cis-DNA elements dictated EPO-R regulation under hypoxia in NSCLC cells. [file 12964_2019_458_MOESM3_ESM.zip › Additional file 3.docx]

**Additional File 3: Supplementary Figures**

**Supplementary Figure. 1. EPO-R protein (A) and mRNA (B) were expressed higher under normoxia in NSCLC cells.** The erythroleukemia line OCIM-1 was used as a positive control. Protein level was determined by Western blots with b-actin included as a loading control; mRNA determined by real-time RT-PCR with cyclophilin as a control. Mean±SEM; *P<0.05, ** P<0.01.

**Supplementary Figure 2. Identification of a cis-DNA elements dictated EPO-R regulation under hypoxia in NSCLC cells.** (A) Sequence analysis of a 200-bp human EPO-R proximal promoter (Genebank accession# M76595.1). Putative binding sites of hypoxia inducible factor (HIF), specificity protein 1 (SP1) and early growth responsive 1 (EGR1) were identified. HRE=hypoxia responsive element. (B) Homologous comparison of human, mouse and dog proximal EPO-R promoter sequences. Putative binding sites of HIF, EGR and SP1 were conserved among human, mouse and dog.

**References**

1. Zhang Q, Moe OW, Garcia JA, Hsia CC. Regulated expression of hypoxia-inducible factors during postnatal and postpneumonectomy lung growth. *Am J Physiol Lung Cell Mol Physiol* 2006, 290:L880-889.

2. Nelson JD, Denisenko O, Bomsztyk K. Protocol for the fast chromatin immunoprecipitation (ChIP) method. *Nat Protoc* 2006, 1:179-185.

3. Zhang Q, Agoston AT, Pham TH, Zhang W, Zhang X, Huo X, et al. Acidic Bile Salts Induce Epithelial to Mesenchymal Transition via VEGF Signaling in Non-Neoplastic Barrett's Cells. *Gastroenterology* 2019, 156:130-144 e110.

4. Mu N, Gu J, Huang T, Zhang C, Shu Z, Li M, et al. A novel NF-kappaB/YY1/microRNA-10a regulatory circuit in fibroblast-like synoviocytes regulates inflammation in rheumatoid arthritis. *Sci Rep* 2016, 6:20059.
