# Supplementary figures and images for: ΗΙF1α, EGR1 and SP1 co-regulate the erythropoietin receptor expression under hypoxia: an essential role in the growth of non-small cell lung cancer cells
Source: Cell Commun Signal. 2019 Nov 21;17:152. doi: 10.1186/s12964-019-0458-8 (PMC6869211; doi:10.1186/s12964-019-0458-8)

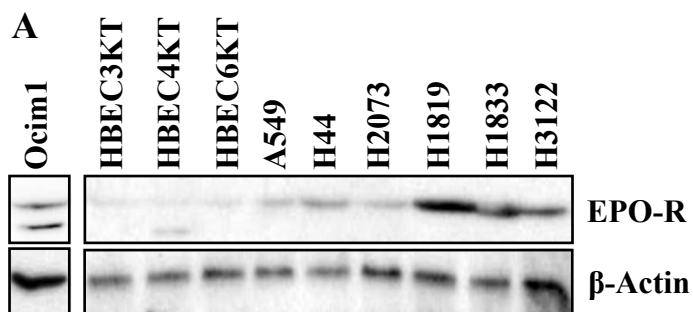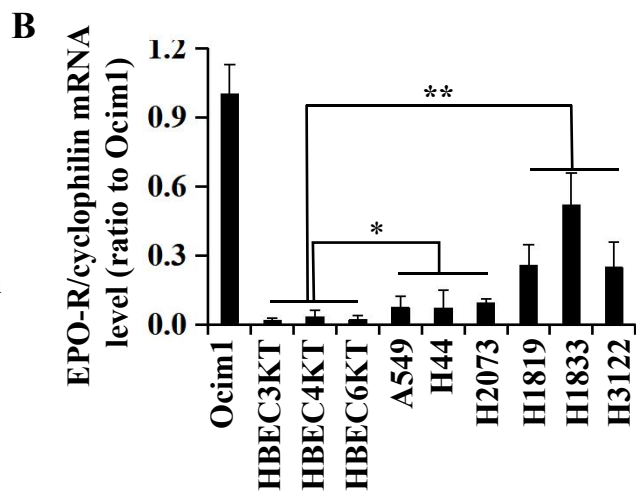

Supplement: Supplementary file 3 — Additional file 3: Figure S1. EPO-R protein (A) and mRNA (B) were expressed higher under normoxia in NSCLC cells. Figure S2. Identification of a cis-DNA elements dictated EPO-R regulation under hypoxia in NSCLC cells. [file 12964_2019_458_MOESM3_ESM.zip › Supplementary Figure 1.pdf]
